# Timing of Dental Implant Placement: The Past, the Present, the Future

**DOI:** 10.1111/jre.70088

**Published:** 2026-02-11

**Authors:** Daniel S. Thoma, Leonardo Mancini, Ronald E. Jung

**Affiliations:** ^1^ Clinic of Reconstructive Dentistry, Center of Dental Medicine University of Zurich Zurich Switzerland

## Abstract

The optimal timing of dental implant placement remains one of the most debated topics in implant dentistry. Patients increasingly demand fast tooth replacement with minimal morbidity and cost; however, clinicians must balance these expectations with biological limitations and long‐term treatment success. Historically, the decision of when to place an implant has reflected the evolution of scientific knowledge, technological advances, and surgical confidence shifting from delayed and cautious protocols to immediate and patient specific strategies. This perspective article, from a clinical standpoint, highlights the evolution of implant timing concepts, current evidence‐based practices, and explores emerging trends that may redefine future paradigms. Today, timing has become a critical factor in implant therapy. “Time truly is everything”. The art lies in knowing when to accelerate treatment to meet patient requests and when to slow down to respect biological healing achieving the delicate balance between efficiency and predictability.

## Graphical abstract

The evolution of dental implant placement timing, from conventional immediate, early, and late protocols based on fixed healing periods to contemporary evidence‐based concepts. It highlights a future shift toward a tailored, patient‐specific approach supported by biologically active materials and artificial intelligence for individualized clinical decision‐making.

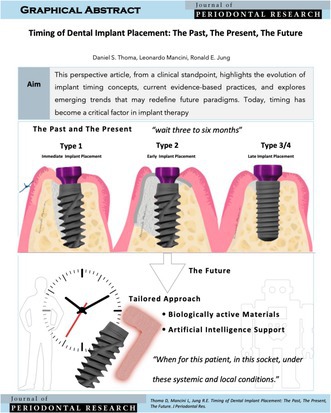

## 1 Introduction

The optimal timing of dental implant placement remains one of the most debated topics in implant dentistry. Patients increasingly demand fast tooth replacement with minimal morbidity and cost; however, clinicians must balance these expectations with biological limitations and long‐term treatment success. Historically, the decision of when to place an implant has reflected the evolution of scientific knowledge, technological advances, and surgical confidence shifting from delayed and cautious protocols to immediate and patient specific strategies.

This perspective article, from a clinical standpoint, highlights the evolution of implant timing concepts, current evidence‐based practices, and explores emerging trends that may redefine future paradigms. Today, timing has become a critical factor in implant therapy.

“*Time truly is everything*”. The art lies in knowing when to accelerate treatment to meet patient requests and when to slow down to respect biological healing achieving the delicate balance between efficiency and predictability.

## 2 The Past: Conventional and Delayed Implant Placement

Modern implantology originated with Schröder's and Brånemark's seminal discovery of osseointegration in the 1960s, describing the direct structural and functional connection between living bone and titanium implants [1, 2].

Early clinical protocols emphasized caution: implants were placed several months after tooth extraction to allow for complete bone healing and minimize infection risk.

This delayed placement approach typically after 3–6 months of post‐extraction healing was regarded as the gold standard for decades [3].

The rationale for delayed placement was multifactorial:

- Infection control: Immediate placement was believed to endanger osseointegration due to microbial contamination.
- Bone remodeling: Complete socket healing was considered necessary for achieving adequate bone density and implant stability.
- Implant design limitations: Early implants featured machined, smooth surfaces with limited mechanical retention, necessitating extended healing for integration [4].

While delayed protocols achieved acceptable long‐term success rates, they involved prolonged treatment durations often exceeding 9–12 months from extraction to final restoration [5].

## 3 The Present: Evidence‐Based Evolution Toward Early and Immediate Placement

Advancements in implant surface technology, biomechanical understanding, and digital planning have transformed traditional concepts of timing.

The International Team for Implantology (ITI) classification system provides a standardized framework dividing implant placement timing into four categories [6]:

1. Type 1: Immediate placement (at the time of extraction).
2. Type 2: Early placement after soft‐tissue healing (4–8 weeks post‐extraction).
3. Type 3: Early placement after partial bone healing (12–16 weeks post‐extraction).
4. Type 4: Late placement after complete bone healing (> 6 months post‐extraction).

### 3.1 Type 1: Immediate Implant Placement

Immediate placement aims to preserve alveolar bone volume, shorten treatment time, and maintain the soft‐tissue envelope [7].

When combined with immediate provisionalization, it offers distinct aesthetic advantages.

However, success depends on rigorous case selection:

- No acute infection or periodontal pathology
- Intact socket walls
- Primary stability (insertion torque ≥ 35 Ncm)
- Controlled grafting of the implant–socket gap

**FIGURE 1:**
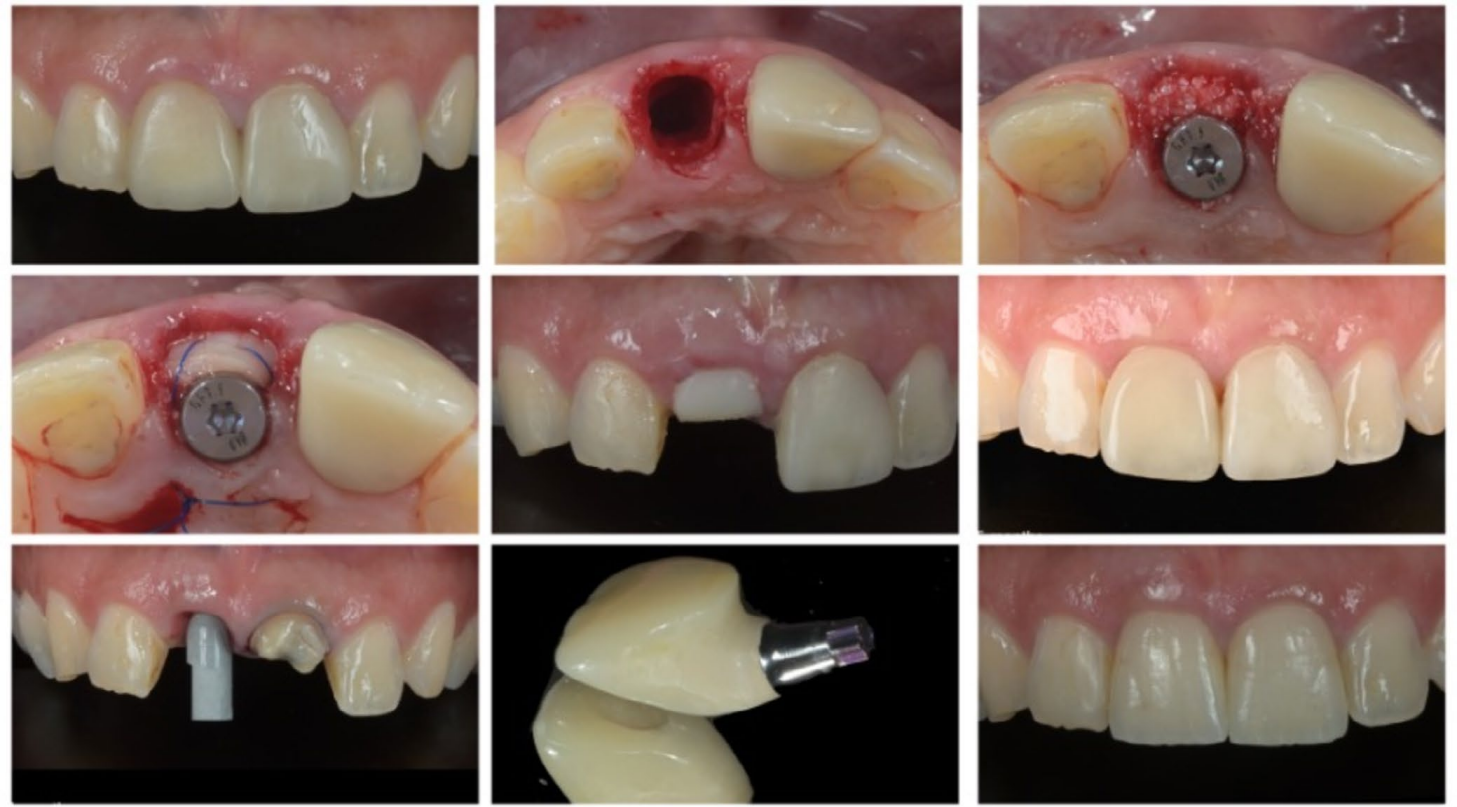
Step‐by‐step immediate implant placement in the anterior region.

When executed properly, survival rates of 95%–98% are reported comparable to delayed protocols [8, 9]. Nonetheless, esthetic complications such as midfacial recession and marginal bone loss remain more frequent in patients with a thin periodontal phenotype [10]. In Figure 1 a representation of a step‐by‐step immediate implant procedure.

### 3.2 Type 2: Early Placement After Soft‐Tissue Healing (4–8 Weeks)

Type 2 placement is performed after initial soft‐tissue closure but before significant bone resorption begins. This approach benefits from a healed mucosal barrier, reducing the risk of bacterial contamination while preserving most of the alveolar ridge contour [11].

The soft‐tissue coverage facilitates tension‐free primary closure and minimizes the likelihood of membrane exposure during guided bone regeneration (GBR). At this stage, socket walls remain largely intact, supporting predictable implant positioning and graft containment [12].

**FIGURE 2:**
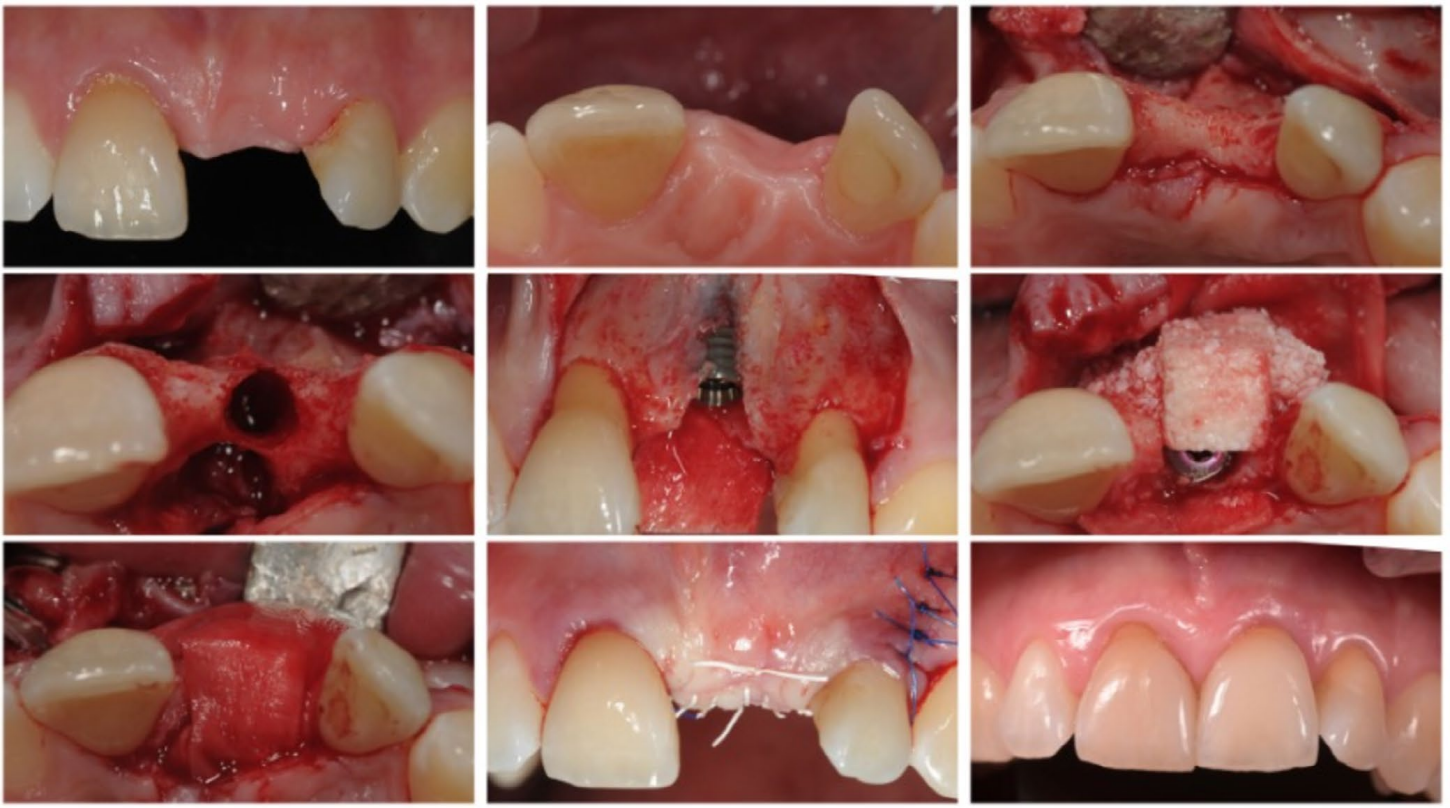
Step‐by‐step early implant placement in the anterior region.

Evidence indicates that Type 2 placement achieves high survival rates (96%–99%) and reduces the risk of mucosal recession compared to immediate placement on the mid to long run, especially in thin periodontal phenotype [6]. It is well suited for sockets with minor infection or limited bone defects where immediate placement is contraindicated [13]. Figure 2 illustrates the step‐by‐step procedure for early implant placement combined with guided bone regeneration.

### 3.3 Type 3 and Type 4 Implant Placement: Early and Late Post‐Extraction Protocols

Type 3 (Early Placement, 12–16 Weeks Post‐Extraction) and Type 4 (Late Placement, > 6 Months Post‐Extraction) represent the later stages of implant placement following tooth extraction, each with distinct clinical indications and biological considerations.

In Type 3 placement, the extraction site shows partial bone fill and early mineralization, providing a stable substrate for implant insertion compared with immediate or early approaches. This timing optimizes primary stability, minimizes infection risk, and limits the degree of alveolar ridge resorption associated with extended healing periods. It is particularly indicated for cases with suboptimal socket conditions, such as thin buccal bone, prior periapical pathology, or sites requiring minor contour augmentation. Clinical studies report survival rates of approximately 97%–99%, demonstrating predictability comparable to delayed protocols [14, 15, 16]. However, moderate ridge reduction may occur during the 3–4‐month healing phase, occasionally requiring minor augmentation at the time of implant placement.

**FIGURE 3:**
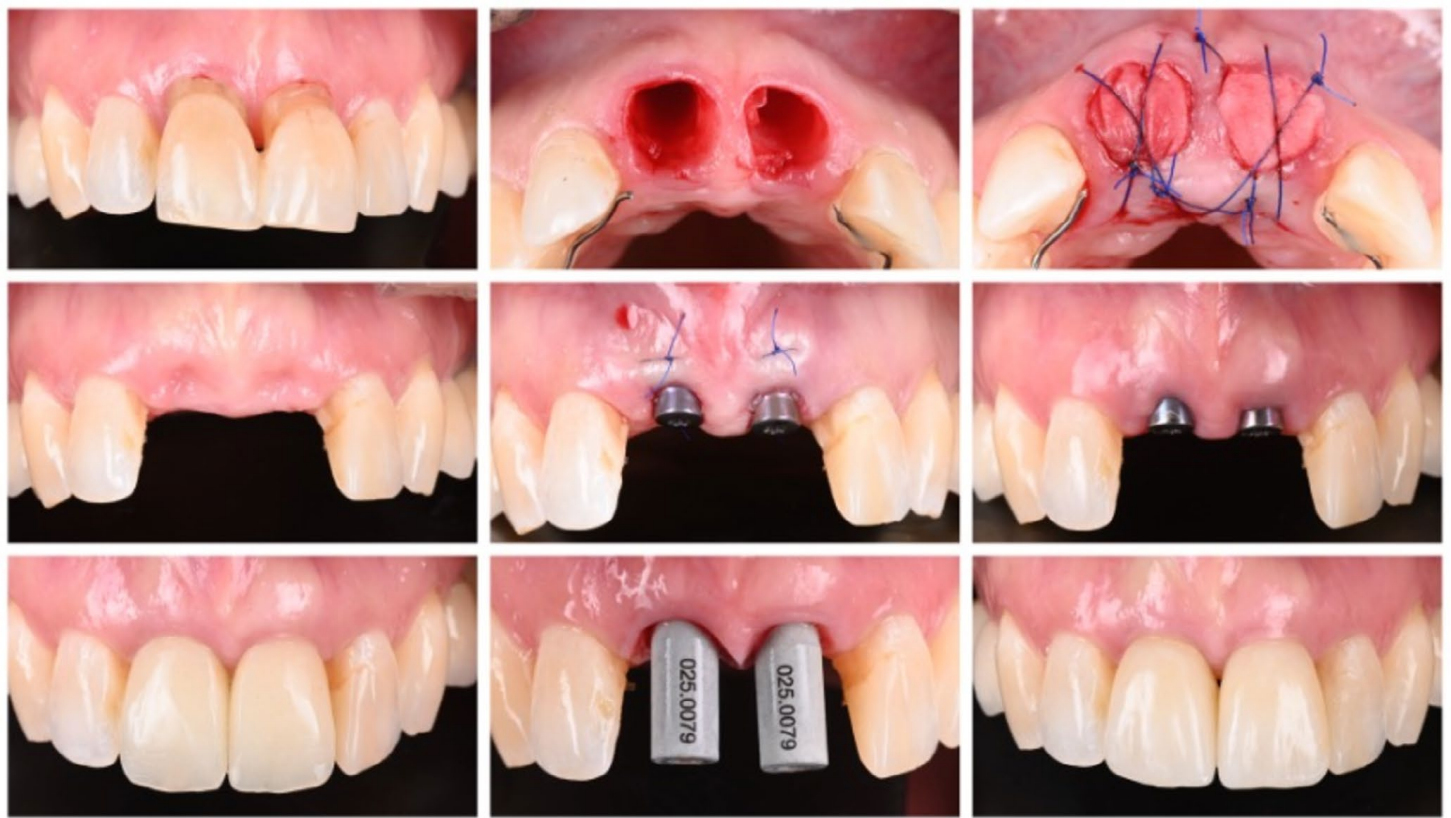
Step‐by‐step Type 4 implant placement in the esthetic region. Alveolar ridge preservation and a Type 4 placement protocol allowed for a flapless approach with micro‐grafting to enhance soft tissue volume at the future prosthetic level.

In contrast, Type 4 placement (late placement after > 6 months of healing) is the most conservative approach, performed after complete bone remodeling. It offers optimal bone quality, mature tissue integration, and allows full assessment of the healed ridge before implant insertion. This protocol is best suited for sites with extensive bone loss, prior infection, or when major augmentation procedures are necessary. Although treatment time is longer and greater ridge resorption may occur, it provides high predictability and excellent soft‐tissue outcomes. Long‐term studies show survival rates exceeding 95%, particularly in posterior regions or low‐esthetic‐demand cases [14, 16, 17]. Figure 3 illustrates a Type 4 case in which ridge preservation was performed, and the stability of the buccal bone allowed the procedure to be carried out using a flapless approach.

Overall, both Type 3 and Type 4 placement protocols yield predictable clinical outcomes when applied appropriately, balancing biological healing, ridge preservation, and surgical timing to ensure long‐term implant success. Table 1 summarizes the different approaches and advantages and disadvantages.

**TABLE 1:** Summary of different time points and their potential advantages and disadvantages.

Timing	Advantages	Disadvantages
Type 1 immediate	Fewer surgical procedures Reduced overall treatment time Immediate temporization	Site morphology can affect anchorage and angulation Phenotype may influence the final outcome Technique sensitive procedure
Type 2 (4–8 weeks)	Increased soft tissue thickness Easier flap management Resolution of any local acute pathology	Site morphology can affect implant position Longer treatment times Grafting procedures are required
Type 3 (12–16 weeks)	Substantial bone fill Easier flap management Dimensional changes have already occurred	Longer treatment times Grafting procedures are still required Unexpected bony wall resorption may occur
Type 4 ≥ 6 months	Stable hard and soft tissue Increased quality of the soft tissue	Longer treatment times Grafting procedures may be required Remaining bone may vary significantly

## 4 Opportunities, Gray Zones, and Clinical Dilemmas

Despite major scientific and technological advances, determining the optimal timing for implant placement in daily clinical practice remains challenging. While the ITI classification provides a valuable and structured framework, it does not fully capture the complexity of real‐world decision‐making. Clinicians are frequently confronted with borderline situations, patient‐specific modifiers, and esthetic risks that extend beyond predefined timing categories.

One of the most debated gray zones involves immediate and early implant placement in extraction sites that are not ideal. Although intact socket walls and the absence of acute infection are commonly prerequisites for Type 1 placement, everyday practice often presents intermediate scenarios. These include sites with limited buccal dehiscences, teeth previously treated endodontically with residual periapical pathology, or sockets with a thin buccal plate measuring less than 1 mm.

Current evidence indicates that such minor defects do not automatically rule out immediate or early implant placement, provided that adequate primary stability can be achieved and appropriate regenerative measures are applied. Nevertheless, it is often difficult to determine at what point the risks become greater than the expected benefits, particularly in esthetically demanding situations. In these situations, clinical judgment, experience, and the ability to manage patient expectations continue to play a central role.

Another persistent challenge relates to patient‐related systemic and behavioral factors that influence healing and long‐term tissue stability. Smoking, insufficient glycemic control, a history of periodontitis, thin, soft‐tissue phenotype, and high smile lines are all well‐recognized risk factors. While their individual effects are reasonably well documented, their combined impact on timing decisions is far less predictable and remains difficult to quantify.

Importantly, successful osseointegration does not necessarily guarantee a stable esthetic outcome over time. For example, immediate implant placement in patients with a thin gingival phenotype and a high smile line may be biologically successful yet carry an increased risk of midfacial recession in the long term. In such cases, early implant placement after soft‐tissue healing (Type 2) often represents a pragmatic compromise slightly extending treatment time in exchange for improved soft‐tissue management and greater esthetic predictability.

## 5 The Future: Toward Personalized and Regenerative Implantology

Future implantology is expected to move beyond rigid timing classifications toward a more individualized and biologically guided decision‐making process. Rather than relying on predefined temporal categories, implant timing will increasingly be tailored to patient‐specific anatomical, biological, and esthetic parameters. This shift is being driven by advances in digital diagnostics, biologically active materials, and artificial intelligence (AI)–supported treatment planning.

Early translational and clinical studies have demonstrated the potential of AI and machine‐learning algorithms to analyze CBCT‐derived data, including bone volume, density, socket morphology, and anatomical constraints. When combined with clinical variables such as smoking status, periodontal history, and systemic health indicators, these models have shown promise for risk stratification and outcome prediction, including implant stability and marginal bone behavior [18, 19]. Although most AI applications remain investigational, initial evidence suggests that such tools may support clinicians in identifying biologically favorable windows for implant placement rather than simply accelerating treatment timelines.

Parallel progress in regenerative and biologically active materials is further reshaping concepts of healing and timing. Growth‐factor–based therapies, bioactive scaffolds, and cell‐modulating biomaterials have demonstrated enhanced osteogenesis, angiogenesis, and soft‐tissue maturation in preclinical and early clinical studies [20, 21]. These approaches are particularly relevant in post‐extraction sites, where accelerated bone formation may reduce the biological limitations traditionally associated with immediate or early implant placement.

Importantly, these developments intersect closely with principles of periodontal regeneration and peri‐implant phenotype modification. Techniques aimed at increasing soft‐tissue thickness such as connective tissue grafting, volume‐stable collagen matrices, and bioactive soft‐tissue scaffolds have been shown to improve peri‐implant tissue stability and reduce the risk of midfacial recession [22, 23]. Emerging biomaterials may further enhance these effects by actively modulating tissue quality rather than merely replacing lost volume.

As these technologies mature, healing time following tooth extraction may increasingly be viewed as a modifiable, treatment‐controlled variable rather than a fixed biological limitation. Future implant timing decisions are therefore likely to be guided less by chronological intervals and more by the current healing status of the tissues, informed by digital diagnostics, regenerative response, and patient‐specific risk profiling.

## 6 Conclusion

Over the next decade, implant placement timing will evolve from a rigid, schedule‐based paradigm to a dynamic, biologically informed, patient‐centered process.

Instead of universally applying rules like *“*wait three to six months,” clinicians will adopt an evidence‐based principle of “as early as safely possible” guided by intraoperative diagnostics, enhanced biomaterials, and real‐time digital assessment.

In essence, the future of implant timing will focus less on when and more on “when for this patient, in this socket, under these systemic and local conditions.”

The evolution will continue toward “faster, smarter, and safer outcomes” anchored in precision rather than convention.

## Funding

The authors have nothing to report.

## Conflicts of Interest

The authors declare no conflicts of interest.

## Data Availability

The data that support the findings of this study are available on request from the corresponding author. The data are not publicly available due to privacy or ethical restrictions.
